# Comprehensive analysis of the prognosis and immune infiltration for CXC chemokines in colorectal cancer

**DOI:** 10.18632/aging.203245

**Published:** 2021-07-07

**Authors:** Xi Yang, Yuanfeng Wei, Feng Sheng, Yirong Xu, Jiao Liu, Ling Gao, Ju Yang, Xinchen Sun, Junxing Huang, Qing Guo

**Affiliations:** 1 Department of Medical Oncology, Cancer Center, West China Hospital, Sichuan University, Chengdu, China; 2 Department of Neurosurgery, Taizhou People’s Hospital, Taizhou, China; 3 Department of Oncology, Taizhou People’s Hospital, Taizhou, China; 4 Department of Pathophysiology, West China School of Basic Medical Sciences and Forensic Medicine, Sichuan University, Chengdu, China; 5 Department of Radiation Oncology, The First Affiliated Hospital of Nanjing Medical University, Nanjing, China

**Keywords:** comprehensive analysis, CXC chemokines, colorectal cancer, immune infiltration

## Abstract

The C-X-C motif (CXC) chemokines are a family of chemotactic molecules that have been identified as potential prognostic markers and prospective therapeutic targets for many kinds of cancer types. Increasing evidence shows that CXC chemokines are associated with the progression of colorectal cancer (CRC); however, the correlations of CXC chemokines with prognostic and immune infiltrates in CRC remain to be clarified. In this study, we analyzed the mRNA expression level, prognostic data and immune infiltrates of CXC chemokines in CRC patients from the Gene Expression Profiling Interactive Analysis, Oncomine, cBioPortal and databases using GeneMANIA, STRING, DAVID 6.8, and TIMER. Our results showed that CXCL1/2/3/4/5/8/9/10/11/13/14/16 were significantly overexpressed in CRC tissues. Furthermore, expression of CXCL1/2/3/9/10/11 was associated with tumor stage in CRC. A significant association was also identified between the co-expression of CXCL16 with EGFR, KRAS and NRAS. In addition, the survival analysis suggested that high CXCL2/3/8/9/10/11/14 expression is correlated with clinical outcomes of CRC patients. Moreover, a significant association was observed between the CXCL8/9/10/11 expression and immune infiltration in colonic and rectal adenocarcinoma. Overall, CXC chemokines are not only implicated as prognostic biomarkers for CRC patients, but may also influence the immune status of CRC tissues.

## INTRODUCTION

Colorectal cancer (CRC), including colon cancer and rectal cancer, is the second most common malignancy and the third cause of cancer-related deaths worldwide [1]. In 2020, approximately 147,950 new cases of CRC were diagnosed, including an estimated 53,200 CRC-related deaths in the United States [2]. Despite significant progress and considerable efforts aimed at the development of multidisciplinary therapies, the quality of life and survival of patients with severe CRC remain unsatisfactory [3]. Furthermore, the detailed molecular mechanisms in CRC pathogenesis are still not fully understood [4]. Therefore, it is necessary to identify prognostic markers and characterize the underlying immune infiltration in CRC to efficiently improve prognosis and individualized treatments.

The C-X-C motif (CXC) chemokines are a group of approximately 50 chemotactic molecules of low-molecular-weight that are known to function as the basic regulators of the directional migration of leukocytes [5]. Chemokines are chemoattractants secreted by leukocytes, tumor cells and other cell types, such as epithelial cells, fibroblasts, and immune cells [6]. These small proteins play a crucial role in many biological processes associated with cancers, such as tumor growth, progression, metastasis, and angiogenesis, and influence therapeutic effect, and patient outcomes [4, 6]. According to the relative spacing of conserved cysteine residues in the N-terminal region of the peptide chain, chemokines have been classified into four broad groups, C, CC, CXC, and CX3C chemokines, in which the X represents any amino acid [4]. As a vital member of the chemokine family of proteins, CXC chemokines have been identified as potential prognostic markers and prospective therapeutic targets in many kinds of cancers, including breast cancer and kidney cancer [3, 7, 8].

To date, sixteen CXC chemokines have been detected in mammalian cells and named to reflect the order of their discovery (CXCL1/2/3/4/5/6/7/8/9/10/11/12/13/14/16/17) [6]. Multiple studies have indicated that CXC chemokines play vital roles in the development and progression of CRC [4, 9]. For instance, CXCL1 has been reported to be associated with CRC progression and metastasis by inducing glycolysis [10]. Additionally, recent studies have indicated that CXCL8 promotes angiogenesis and cell migration in CRC [11]. Furthermore, Lin et al. reported that CXCL14 participates in the progression of CRC and is encoded by a candidate cancer suppressor gene [12]. However, the role of CXC chemokine members in CRC tumor progression and development is highly complex. Further studies are required to investigate the role of the intricate CXC chemokine network in tumor development to provide new insights for therapeutic applications in patients with CRC. In this study, we conducted a comprehensive bioinformatics analysis of the expression and mutational activation of CXC chemokine members and their link with potential prognosis and immune infiltrates in CRC patients.

## MATERIALS AND METHODS

### Ethics statement

The collection of colorectal tissues was approved by the Ethics Committee of the Taizhou People’s Hospital, Suzhou, China (project no. KY 202005401), and was conducted according to the relevant guidelines. The datasets were obtained from the relevant published literature.

### Oncomine analysis

The transcription levels of CXC chemokines in different cancers were downloaded from a cancer microarray database (Oncomine, https://www.oncomine.org). The differences in expression levels of CXC chemokines at the mRNA level in CRC and corresponding normal tissues were evaluated by Student’s *t*-test. The cutoffs for the identification of differentially expressed genes were set as *P* < 0.0001 and a fold change in expression ≥2.

### Gene expression profiling interactive analysis (GEPIA) dataset

GEPIA, which is an interactive web server, was used to assess the RNA sequencing expression, co-expression, and survival data of 8, 587 normal samples and 9, 736 tumors from the Genotype Tissue Expression projects and The Cancer Genome Atlas (TCGA) according to the standard processing pipeline (http://gepia.cancer-pku.cn). GEPIA was used to compare the mRNA expression levels of CXC chemokines between CRC and normal samples. The correlation between CXC chemokines expression and patient prognosis in colon adenocarcinoma (COAD), rectal adenocarcinoma (READ), and colon and rectal adenocarcinoma (COAD + READ) were analyzed in terms of disease-free survival (DFS), overall survival (OS), and the tumor stage. Moreover, the relationships between CXC chemokine expression and EGFR, KRAS and NRAS of COAD, READ, and COAD + READ were evaluated.

### Immunohistochemistry (IHC) analysis

All samples were obtained from CRC patients who underwent initial surgical resection in Taizhou People’s Hospital. CRC samples and adjacent noncancerous colorectal tissues were collected for IHC analysis using the following primary detection antibodies: anti-CXCL2 (Bioss, Cat#bs200208R, China), anti-CXCL3 (Bioss, Cat#bs2547R), anti-CXCL8 (PTG, Cat#27095-1-AP, China), anti-CXCL9 (PTG, Cat#22355-1-AP, China), anti-CXCL10 (PTG, Cat#10937-1-AP, China), anti-CXCL11 (PTG, Cat#10707-1-AP, China), and anti-CXCL14 (PTG, Cat#10468-1-AP, China). Immunohistochemistry analysis was used following the manufacturer’s protocol.

### cBioPortal for cancer genomics analysis

The CRC datasets (TCGA, Firehose legacy) involving data from 640 cases with pathology reports were selected for further analysis of CXC chemokines using cBioPortal (https://www.cbioportal.org/). The genomic profiles included protein expression z-scores (RPPA), mutations, mRNA expression z-scores (RNA Seq V2 RSEM), and putative copy-number alterations from genomic identification of significant targets in cancer (GISTIC). Moreover, co-expression was assessed according to cBioPortal and using the R package “ggcorrplot”.

### GeneMANIA and STRING

GeneMANIA, which is a web-based dataset, was used to analyze the internal associations of gene sets (https://genemania.org/). STRING is a database of predicted protein-protein interactions, including direct (physical) and indirect (functional) associations (https://string-db.org/). Therefore, the interactions of CXCL1/2/3/4/5/6/7/8/9/10/11/12/13/14/16/17 were analyzed at the gene and protein expression levels by GeneMANIA and STRING.

### Database for annotation, visualization and integrated discovery (DAVID) analysis

As a bioinformatics resource, DAVID (version 6.8) is an open database focusing on gene functional annotations and enrichment pathway analysis (https://david.ncifcrf.gov/home.jsp). In our study, DAVID was used to annotate the cellular component (CC), gene ontology (GO) terms for biological process, Kyoto Encyclopedia of Genes and Genomes (KEGG) pathway, and molecular function categories (MF) of CXCL1/2/3/4/5/6/7/8/9/10/11/12/13/14/16/17.

### KEGG analysis

The KEGG (https://www.kegg.jp/) is a database resource for understanding the high-level functions of biological systems. The signaling pathway enrichments of CXCL1/2/3/4/5/6/7/8/9/10/11/12/13/14/16/17 were identified and adjusted according to KEGG and Cytoscape.

### Tumor IMmune Estimation Resource (TIMER)

TIMER2.0 is a comprehensive resource for the systematic analysis of immune infiltrates across different cancer types from TCGA (http://timer.cistrome.org/) [13, 14]. We identified the association between CXCL2/3/8/9/10/11/14 expression in COAD and READ and the abundances of six immune infiltrates, including CD4^+^ T cells, CD8^+^ T cells, B cells, macrophages, dendritic cells (DCs), and neutrophils. TIMER2.0 generates a heatmap table of the Spearman’s correlations between the expression of CXCL2/3/8/9/10/11/14 and the abundance of immune cell type as well as its subtypes across COAD and READ. Moreover, correlations between genetic markers of tumor-infiltrating immune cells and CXCL2/3/8/9/10/11/14 expression were evaluated using correlation modules (Gene_Corr). According to TIMER2.0, the positive correlation is defined as *P* < 0.05, rho > 0; the negative correlation is defined as *P* < 0.05, rho < 0; not significant is defined as *P* ≥ 0.05.

## RESULTS

### Transcription levels of CXC chemokines in patients with CRC

**Figure 1:**
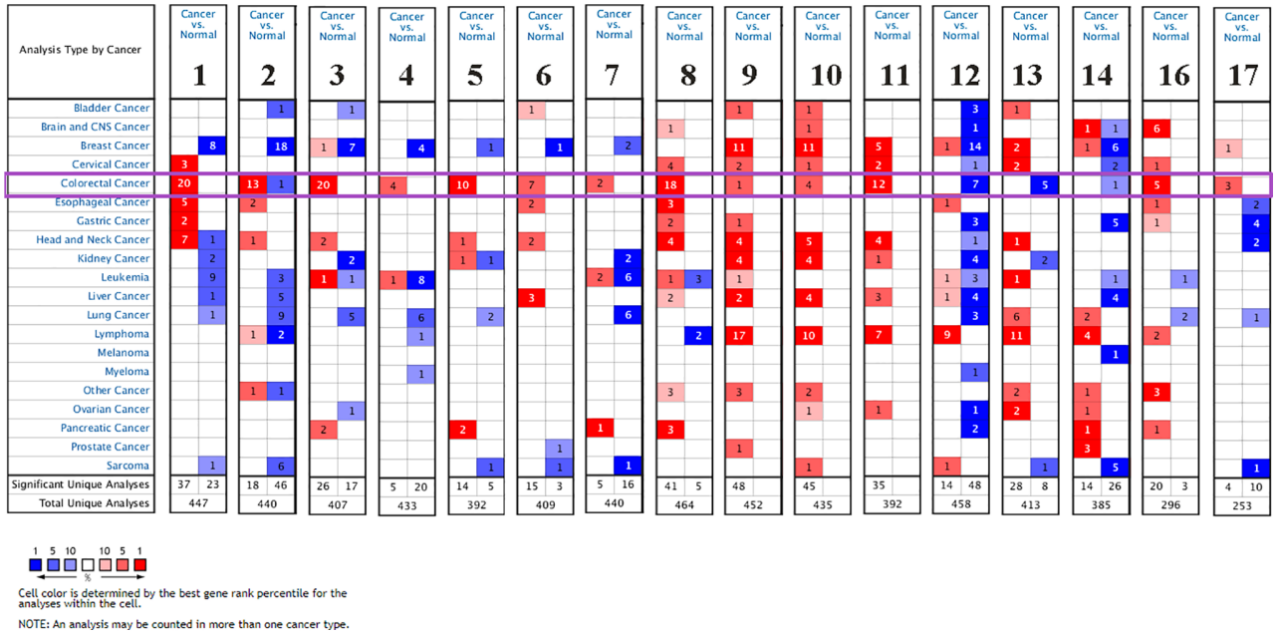
**The mRNA expression levels of CXC chemokines in different types of cancers (Oncomine).** The figure presents the numbers of datasets with statistically significant mRNA over-expression (red) or downregulated expression (blue) of CXC chemokines.

The transcription levels of CXC chemokine family members (excluding CXCL15) were assessed in human cancers. As indicated as Table 1 and Figure 1, we used Oncomine to compare the mRNA expression levels of CXC chemokines in CRC with those in normal samples. This analysis showed that the transcription levels of CXCL1/2/3/4/5/6/7/8/9/10/11/16/17 were significantly upregulated in CRC patients. However, the mRNA expression levels of CXCL12/13/14 were significantly downregulated in CRC patients.

**Table 1:** The significant changes of CXCL expression in transcription level between different types of CRC and normal colorectal tissues (Oncomine database).

	**Type of colorectal cancer**	**Normal**	**Fold change**	***P* Value**	***t* Test**	**Dataset**
**CXCL1**	Colon adenocarcinoma	Colon	12.328	7.62E-9	7.379	Notterman colon
	Colon carcinoma	Colon	5.695	2.71E-5	7.410	Zou colon
	Colorectal adenoma epithelia	Colorectal	2.641	4.71E-8	6.147	Gaspar colon
	Colon carcinoma epithelia	Colon	13.052	8.47E-12	21.652	Skrzypczak colorectal 2
	Colon mucinous adenocarcinoma	Colon	9.298	2.14E-13	10.386	TCGA colorectal
**CXCL2**	Colon adenoma	Colon	12.924	7.11E-10	15.037	Skrzypczak colorectal 2
	Colorectal carcinoma	Colorectal	2.821	2.82E-7	6.75	Grauden colon
	Rectal adenocarcinoma	Rectal	7.368	2.82E-33	16.274	Gaedcke colorectal
	Colorectal carcinoma	Colorectal	10.114	9.83E-12	9.634	Skrzypczak colorectal
	Rectal adenocarcinoma	Rectal	8.625	1.62E-5	7.632	Kaiser colon
**CXCL3**	Colorectal adenoma epithelia	Colorectal	2.117	5.34E-8	6.255	Gaspar colon
	Colon carcinoma epithelia	Colon	9.918	1.89E-9	13.837	Skrzypczak colorectal 2
	Rectal adenocarcinoma	Rectal	10.56	3.02E-6	8.107	Kaiser colon
	Colon adenocarcinoma	Colon	4.193	4.28E-16	9.795	Ki colon
	Colorectal carcinoma	Colorectal	11.017	5.55E-12	10.192	Skrzypczak colorectal
**CXCL4**	Colon carcinoma	Colon	5.228	5.84E-10	15.615	Skrzypczak colorectal 2
	Colorectal adenocarcinoma	Colorectal	2.704	9.38E-11	7.697	Skrzypczak colorectal
	Colon adenoma	Colon	5.012	1.96E-11	8.964	Sabates-Bellver colon
**CXCL5**	Rectosigmoid adenocarcinoma	rectosigmoid	11.693	8.34E-14	16.245	TCGA colorectal
	Colorectal carcinoma	Colorectal	11.504	2.50E-11	8.148	Skrzypczak colorectal
	Colon adenocarcinoma	Colon	3.682	1.19E-10	8.196	Kaiser colon
	Rectal adenocarcinoma	Rectal	12.096	8.89E-23	12.435	Gaedcke colorectal
	Colon adenoma	Colon	4.184	5.17E-5	4.637	Sabates-Bellver colon
**CXCL6**	Colorectal carcinoma	Colorectal	4.156	3.39E-10	7.401	Skrzypczak colorectal
	Colon adenocarcinoma	Colon	2.428	7.22E-9	6.53	Ki colon
	Colon carcinoma epithelia	Colon	6.937	5.37E-7	16.403	Skrzypczak colorectal 2
	Rectal adenocarcinoma	Rectal	3.095	5.73E-17	10.672	Gaedcke colorectal
	Colon mucinous adenocarcinoma	Colon	5.107	4.92E-8	7.029	TCGA colorectal
**CXCL7**	Colorectal carcinoma	Colorectal	14.815	4.41E-10	7.328	Hong colorectal
	Colorectal carcinoma	Colorectal	6.016	1.49E-6	5.489	Skrzypczak colorectal
**CXCL8**	Colon mucinous adenocarcinoma	Colon	17.688	6.96E-10	13.138	Kaiser colon
	Colon adenocarcinoma	Colon	3.617	2.66E-5	4.518	Alon colon
	Colon adenocarcinoma	Colon	4.661	2.99E-5	4.584	Notterman colon
	Colon adenoma	Colon	6.531	4.39E-9	14.898	Skrzypczak colorectal 2
	Rectosigmoid adenocarcinoma	rectosigmoid	18.411	2.83E-9	8.982	TCGA colorectal
**CXCL9**	Colon carcinoma epithelia	Colon	7.004	2.28E-7	9.52	Skrzypczak colorectal 2
**CXCL10**	Colorectal carcinoma	Colorectal	6.673	3.40E-10	8.062	Skrzypczak colorectal
	Colon adenocarcinoma	Colon	2.574	2.13E-8	6.061	Ki colon
	Colon carcinoma epithelia	Colon	17.521	2.44E-7	9.735	Skrzypczak colorectal 2
	Rectal adenocarcinoma	Rectal	3.816	1.08E-17	10.009	Gaedcke colorectal
	Colon adenocarcinoma	Colon	2.694	0.012	2.39	Notterman colon
**CXCL11**	Colon carcinoma epithelia	Colon	29.164	9.03E-13	30.893	Skrzypczak colorectal 2
	Colorectal carcinoma	Colorectal	10.483	9.20E-14	9.523	Skrzypczak colorectal
	Colorectal carcinoma	Colorectal	2.112	2.00E-5	4.957	Graudens colon
	Rectal adenocarcinoma	Rectal	12.5	3.15E-7	8.481	Sabates-Bellver colon
	Colorectal carcinoma	Colorectal	5.68	1.21E-8	7.927	Hong colorectal
**CXCL12**	Colon carcinoma epithelia	Colon	1.248	0.007	3.131	Skrzypczak colorectal 2
**CXCL14**	Rectosigmoid adenocarcinoma	rectosigmoid	2.585	0.021	3.666	TCGA colorectal
**CXCL16**	Colorectal carcinoma	Colorectal	2.636	2.08E-18	13.425	Hong colorectal
	Colon adenoma	Colon	2.382	1.42E-13	10.197	Sabates-Bellver colon
	Colon carcinoma epithelia	Colon	3.244	1.64E-5	6.191	Skrzypczak colorectal 2
	Colon mucinous adenocarcinoma	Colon	2.443	1.04E-5	6.809	Kaiser colon
	Colorectal adenocarcinoma	Colorectal	1.822	6.21E-11	8.45	Skrzypczak colorectal
**CXCL17**	Rectosigmoid adenocarcinoma	rectosigmoid	2.664	1.25E-5	7.31	TCGA colorectal
	Colorectal carcinoma	Colorectal	1.301	7.56E-5	4.067	Skrzypczak colorectal
	Rectosigmoid adenocarcinoma	rectosigmoid	1.237	0.013	2.552	Kaiser colon
	Rectal adenocarcinoma	Rectal	1.35	2.72E-5	4.316	Gaedcke colorectal
	Colon carcinoma epithelia	Colon	1.248	0.032	2.242	Skrzypczak colorectal 2

The transcription levels of CXCL1 in colorectal carcinoma were higher than those in colorectal tissues in all 10 datasets. In the Notterman colon dataset, the mRNA expression levels of CXCL1 in colon adenocarcinoma were increased (fold change = 12.328, *P* = 7.62E-9). In the Skrzypczak colorectal 2 dataset, CXCL2 was overexpressed at the mRNA level in colon adenoma (fold change = 12.924, *P* = 7.11E-10), colon epithelial carcinoma (fold change = 7.451, *P* = 2.29E-10), colon epithelial adenoma (fold change = 10.404, *P* = 3.08E-7), and colon carcinoma (fold change = 8.527, *P* = 6.09E-9). In the Gaspar colon dataset, CXCL3 was overexpressed in colon epithelial adenoma (fold change = 2.117, *P* = 5.34E-8) compared with that in colon tissues. In the Skrzypczak colorectal 2 dataset, CXCL4 was overexpressed in colon carcinoma (fold change = 5.228, *P* = 5.84E-10) and colon epithelial adenoma (fold change = 3.165, *P* = 8.43E-7). The mRNA levels of CXCL5 in rectosigmoid adenocarcinoma (fold change = 11.693, *P* = 8.34E-14), colon adenocarcinoma (fold change = 2.739, *P* = 3.60E-22), colon mucinous adenocarcinoma (fold change = 23.08, *P* = 9.97E-11), and rectal adenocarcinoma (fold change = 2.538, *P* = 3.20E-13) were significantly higher in TCGA dataset. In addition, analysis of the Skrzypczak colorectal dataset indicated the mRNA expression levels of CXCL6 were significantly unregulated in patients with colorectal carcinoma (fold change = 4.156, *P* = 3.39E-10).

A similar trend was found in the Hong and Skrzypczak colorectal datasets, with mRNA expression levels of CXCL7 found to be significantly higher in CRC (fold change = 14.815, *P* = 4.41E-10 and fold change = 6.016, *P* = 1.49E-6, respectively). The results from nine datasets indicated that the mRNA expression levels of CXCL8 were significantly upregulated in patients with colorectal carcinoma. In the Skrzypczak colorectal 2 dataset, CXCL9 was overexpressed in colon epithelial carcinoma (fold change = 7.004, *P* = 2.28E-7) compared with that in non-cancerous colon tissues. Furthermore, in the Skrzypczak colorectal 2 dataset, the mRNA expression levels of CXCL10 were upregulated in colon carcinoma (fold change = 6.673, *P* = 3.40E-10). In the Skrzypczak colorectal 2 dataset, the mRNA levels of CXCL11 were increased in colon epithelial carcinoma (fold change = 29.164, *P* = 9.03E-13), colon carcinoma (fold change = 32.137, *P* = 3.09E-9), colon adenoma (fold change = 4.306, *P* = 6.60E-7), and colon epithelial adenoma (fold change = 5.543, *P* = 2.83E-5). In the Hong colorectal dataset, CXCL16 was upregulated in colon carcinoma (fold change = 2.636, *P* = 2.08E-18). In addition, the results from TCGA colorectal dataset indicated that the mRNA expression levels of CXCL17 were significantly higher in rectosigmoid adenocarcinoma (fold change = 2.664, *P* = 1.25E-5), rectal mucinous adenocarcinoma (fold change = 3.492, *P* = 1.60E-5), and colon mucinous adenocarcinoma (fold change = 4.178, *P* = 2.10E-7).

### Expression levels and co-expression analysis of CXC chemokines in patients with CRC

**Figure 2:**
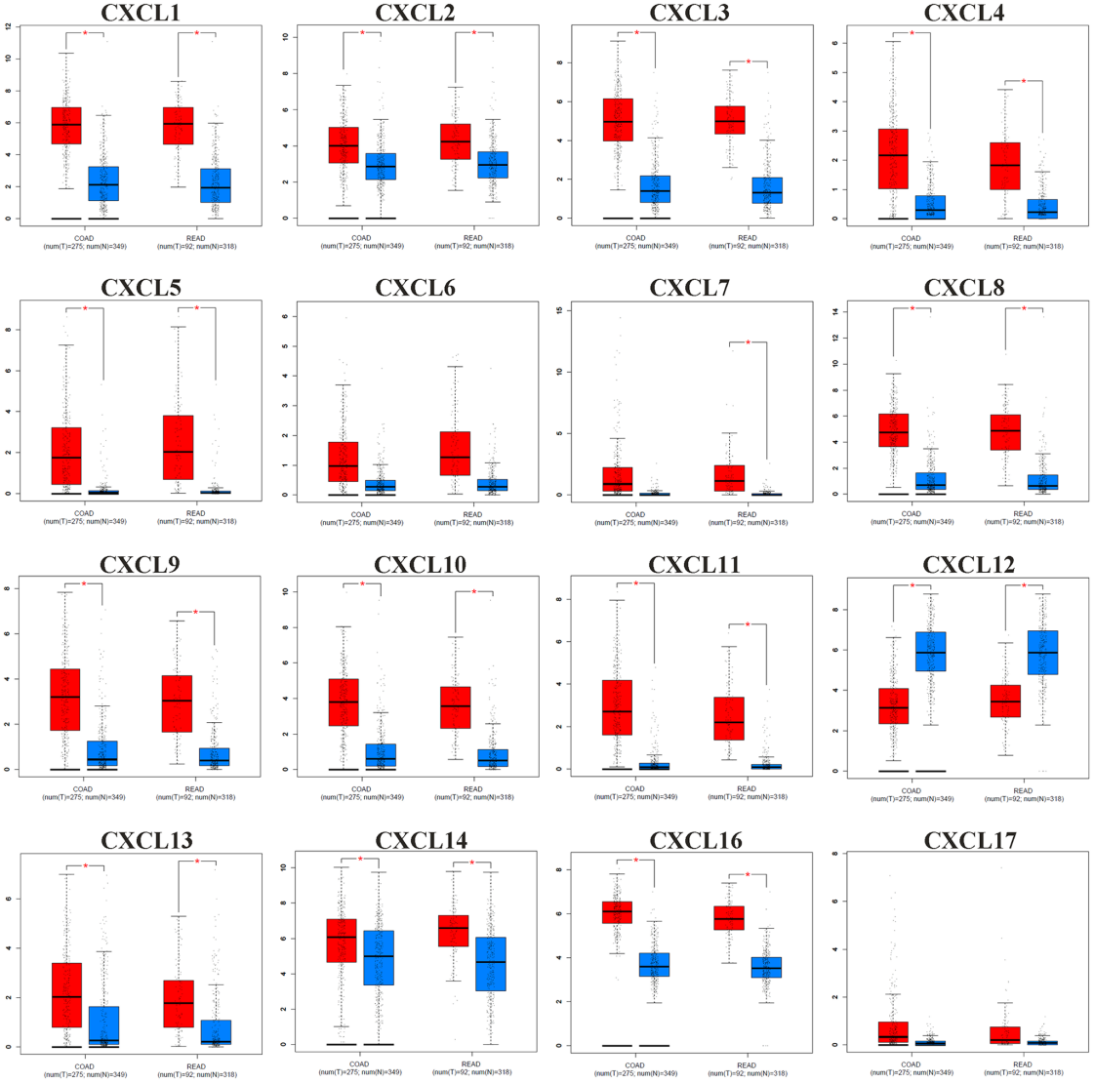
**The mRNA expression pattern of CXC chemokines (CXCL1/2/3/4/5/6/7/8/9/10/11/12/13/14/16/17) from GEPIA between CRC tissues (red) and normal tissues (blue).** The *P*-value was set at 0.05, ^*^ indicate that the results are statistically significant.

The comparative analysis of the CXC mRNA expression in normal vs. COAD and READ tissues was conducted according to the information from the GEPIA database. As shown as Figure 2, the mRNA expression levels of CXCL1/2/3/4/5/8/9/10/11/13/14/16 were significantly upregulated in COAD and READ tissues vs. normal tissue, while CXCL12 expression was downregulated. In addition, the results shown in Supplementary Table 1 revealed significant associations in COAD + READ between tumor stage and expression of CXCL1 (*P* = 0.0345), CXCL2 (*P* = 0.0156), CXCL3 (*P* = 0.0344), CXCL9 (*P* = 0.0122), CXCL10 (*P* = 0.0108), and CXCL11 (*P* = 0.0227) expression.

We also analyzed potential correlations of CXCL1/2/3/4/5/6/7/8/9/10/11/12/13/14/16/17 co-expression with EGFR, KRAS and NRAS according to the mRNA expression levels using the GEPIA database for CRC. The results shown in Table 2 indicated that EGFR expression was significantly correlated with the expression of CXCL3, CXCL12 and CXCL16 in COAD + READ. In addition, a significant association was identified between the expression of KRAS and CXCL16 in COAD + READ. Furthermore, NRAS expression was significantly correlated with the expression of CXCL14, CXCL16 and CXCL17 in COAD + READ.

**Table 2:** The co-expression of different expressed CXC chemokines with EGFR, KRAS and NRAS of CRC patients (GEPIA).

	***P*-value (EGFR)**			***P*-value (KRAS)**			***P*-value (NRAS)**		
	**COAD + READ**	**COAD**	**READ**	**COAD + READ**	**COAD**	**READ**	**COAD + READ**	**COAD**	**READ**
**CXCL1**	0.52	0.55	0.88	0.3	0.87	**0.025**	0.54	0.98	0.063
**CXCL2**	0.099	0.11	0.78	0.48	0.45	0.062	0.41	0.57	0.49
**CXCL3**	**0.011**	0.11	0.96	0.48	0.48	**0.012**	0.97	0.6	0.15
**CXCL4**	0.24	0.21	0.65	0.62	0.59	0.7	0.5	0.68	0.83
**CXCL5**	0.42	0.91	**0.041**	0.94	0.74	0.89	0.81	0.84	0.78
**CXCL6**	0.19	0.31	0.28	0.3	0.29	0.82	0.061	**0.037**	0.99
**CXCL7**	0.35	0.4	0.73	0.49	0.48	0.64	0.21	0.26	0.54
**CXCL8**	0.79	0.85	0.39	0.081	0.46	0.21	0.15	0.12	0.65
**CXCL9**	0.47	0.81	**0.032**	0.79	0.64	0.82	0.68	0.83	0.27
**CXCL10**	0.87	0.73	0.25	0.78	0.7	0.76	0.72	0.85	0.27
**CXCL11**	0.65	0.52	0.26	1	0.79	0.96	0.82	0.79	0.53
**CXCL12**	**0.0045**	**0.019**	0.068	0.4	0.21	0.98	0.13	0.067	0.78
**CXCL13**	0.6	0.35	**0.046**	0.74	0.83	0.42	0.098	0.088	0.79
**CXCL14**	0.28	0.29	0.79	0.51	0.2	0.92	**0.0028**	0.**002**	0.62
**CXCL16**	**1.10E-10**	**4.70E-07**	**1.50E-07**	**0.0086**	**0.014**	0.055	**0.0028**	**0.027**	**0.0047**
**CXCL17**	0.52	0.94	0.2	0.96	0.64	0.77	**0.0047**	0.78	0.077

### Association of CXC chemokine expression with the prognosis of CRC

**Figure 3:**
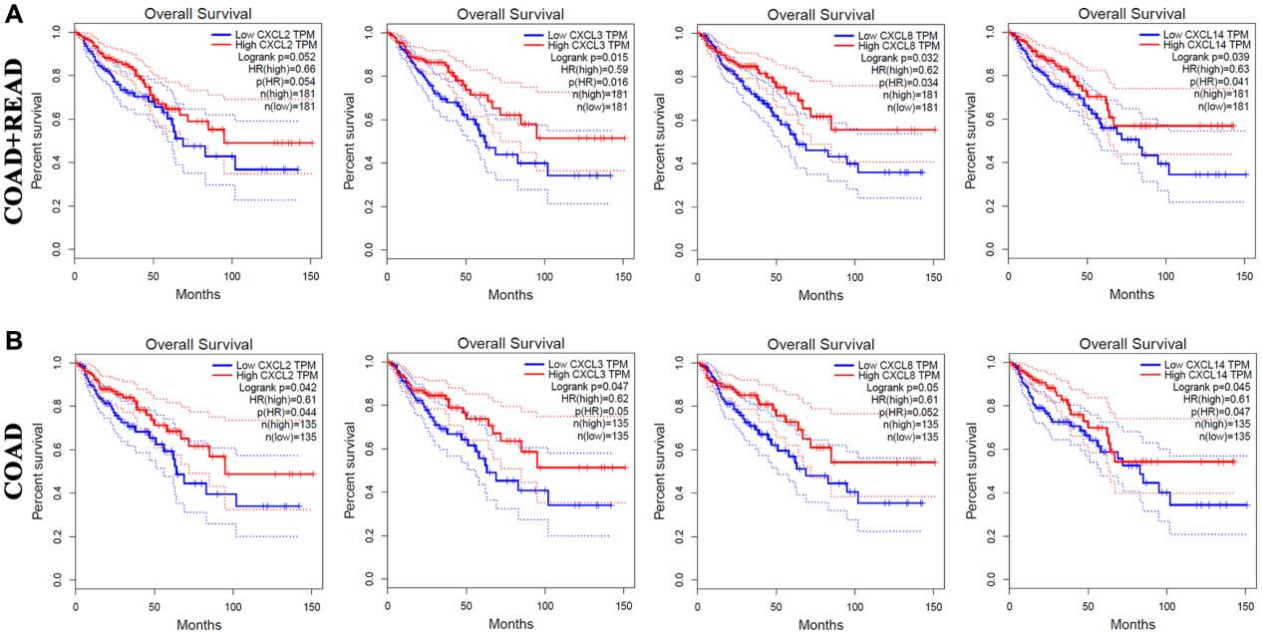
**The prognostic value of different expressed CXC chemokines in CRC patients in OS (GEPIA).** The OS curve of CXCL2/3/8/14 in (**A**) COAD + READ and (**B**) COAD.

Using GEPIA, we also analyzed the correlation between CXC chemokine expression and the OS and DFS of patients with CRC (Supplementary Table 2). The OS curves shown in Figure 3A demonstrated that high mRNA expression levels of CXCL2 (*P* = 0.052), CXCL3 (*P* = 0.015), CXCL8 (*P* = 0.032), and CXCL14 (*P* = 0.039) were correlated with longer OS in 181 CRC patients (COAD + READ). As illustrated as Figure 3B, increased mRNA expression levels of CXCL2 (*P* = 0.042), CXCL3 (*P* = 0.047), CXCL8 (*P* = 0.05), and CXCL14 (*P* = 0.045) were significantly correlated with longer OS in 145 COAD patients. These results indicated that CXCL2/3/8/14 are independent risk factor for OS among CRC patients.

**Figure 4:**
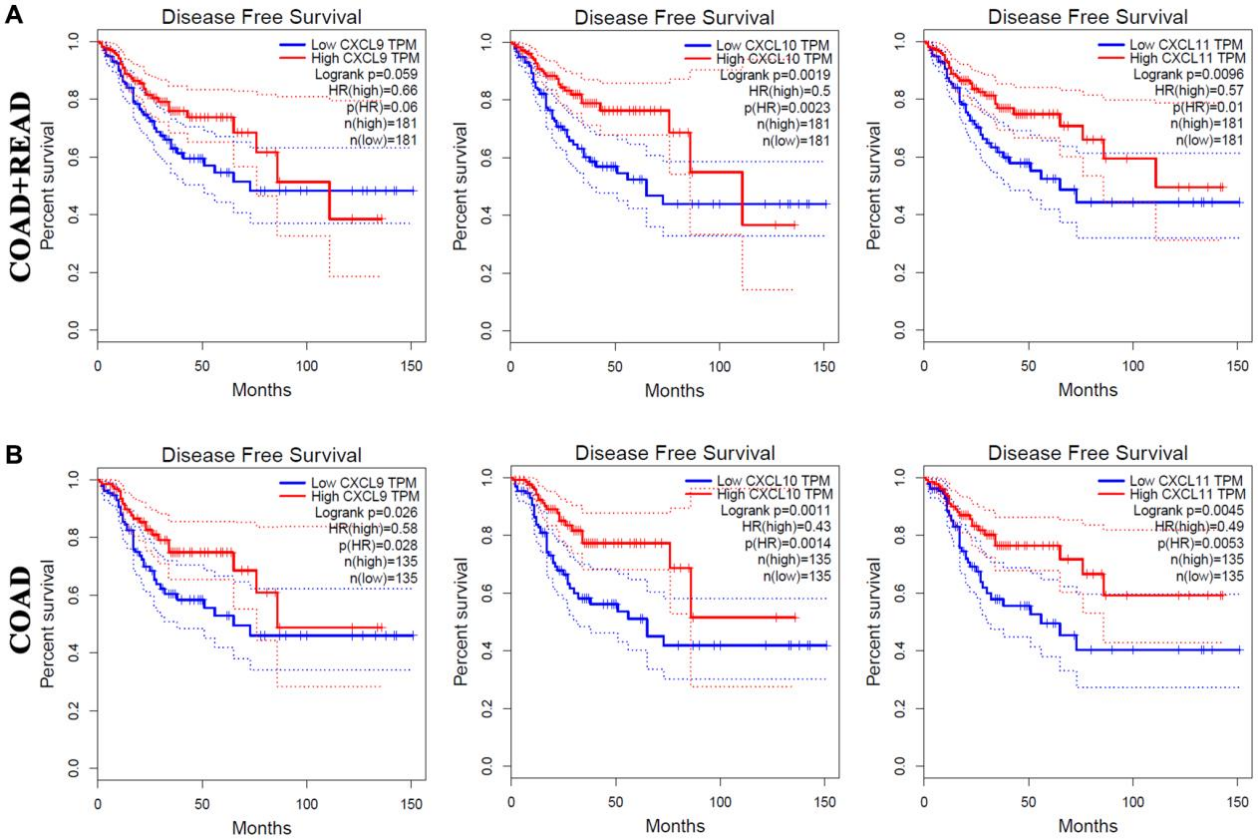
**The prognostic value of different expressed CXC chemokines in CRC patients in DFS (GEPIA).** The DFS curve of CXCL9/10/11 in (**A**) COAD + READ and (**B**) COAD.

The correlations of CXC chemokine expression with the DFS of CRC patients were also investigated (Figure 4.) As shown in in Figure 4A, increased mRNA expression levels of CXCL9 (*P* = 0.059), CXCL10 (*P* = 0.0019), and CXCL11 (*P* = 0.0096) were associated with longer DFS in COAD + READ patients. Moreover, high mRNA levels of CXCL9 (*P* = 0.026), CXCL10 (*P* = 0.0011), and CXCL11 (*P* = 0.0045) were significantly associated with longer DFS in COAD patients (Figure 4B).

### IHC analysis

**Figure 5:**
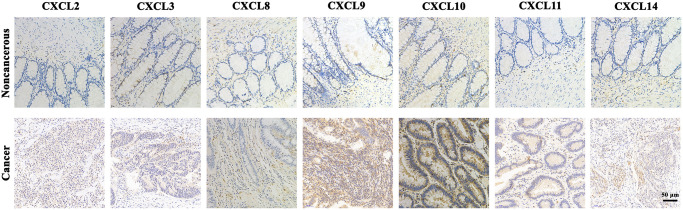
The expression protein of CXCL2/3/8/9/10/11/14 in CRC tissues and noncancerous tissues (IHC).

As shown in Figure 5, IHC analysis of CXCL2/3/8/9/10/11/14 proteins revealed overexpression of CXCL2/3/8/9/10/11/14 proteins in CRC tissues compared with the adjacent noncancerous tissues.

### Alteration, co-expression and interaction analyses of CXC chemokines at the gene and protein levels in patients with CRC

**Figure 6:**
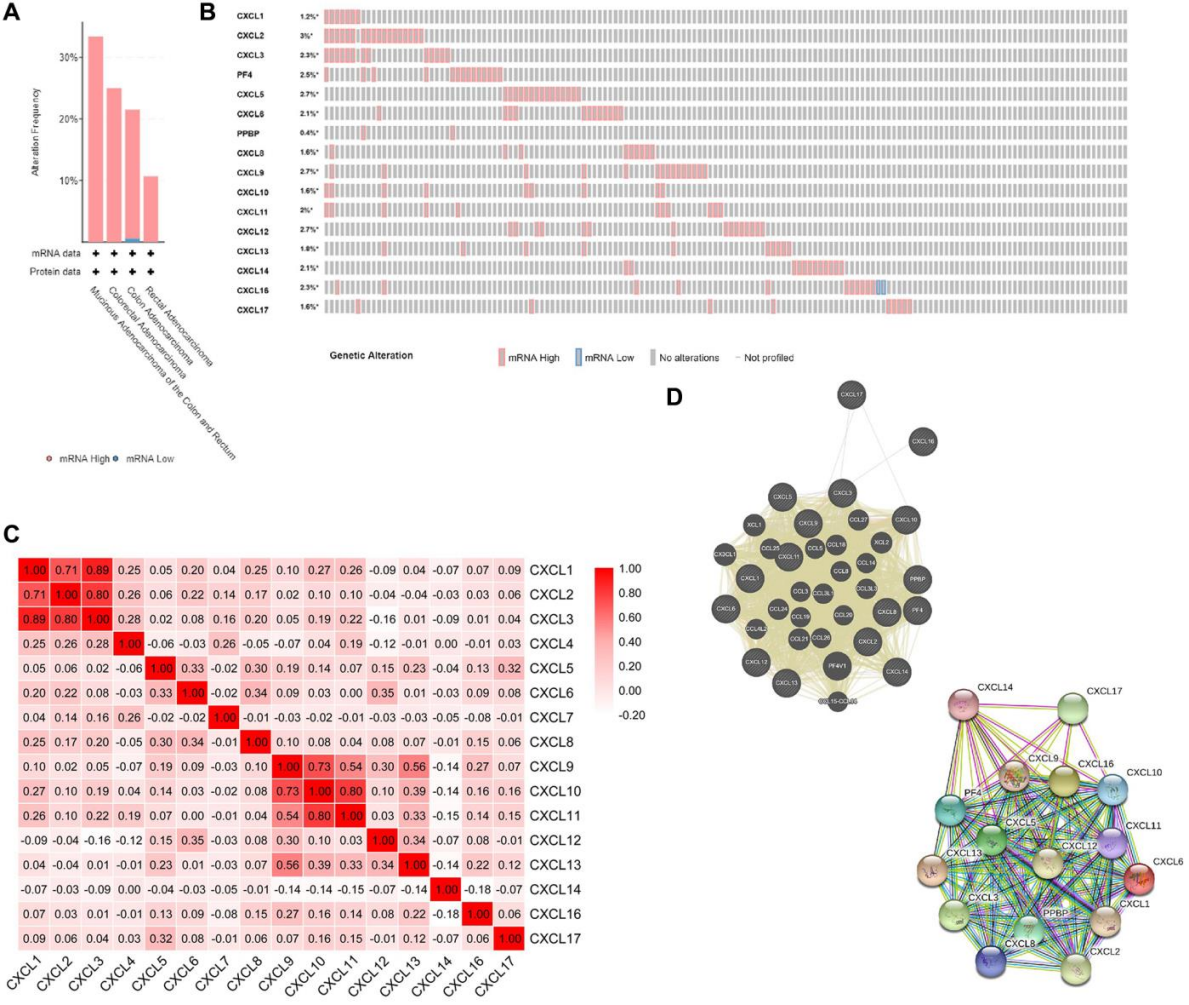
**CXC chemokines gene expression and mutation analysis in CRC (cBioPortal).** (**A**) Summary of alterations in CXC chemokines (cBioPortal). (**B**) OncoPrint visual summary of alteration on a query of CXC chemokines (cBioPortal). (**C**) Correction between different CXC chemokines in CRC (cBioPortal). (**D**) Gene-gene interaction network (GeneMANIA) and protein-protein interaction network (STRING) among CXC chemokines.

We investigated the alterations and correlations of CXC chemokine expression at the gene and protein levels using the cBioPortal database for CRC (TCGA, Firehose legacy). CXC chemokine expression was were altered in 131 of 640 (21%) of CRC samples. As shown as in Figure 6A and 6B, we observed alterations in the expression of CXCL1/2/3/4/5/6/7/8/9/10/11/12/13/14/16/17 in 1.2%, 3%, 2.3%, 2.5%, 2.7%, 2.1%, 0.4%, 1.6%, 2.7%, 1.6%, 2%, 2.7%, 1.8%, 2.1%, 2.3%, and 1.6% of the queried CRC samples, respectively. We also analyzed the co-expression patterns among the chemokines (CXCL1/2/3/4/5/6/7/8/9/10/11/12/13/14/16/17) at the mRNA level using the cBioPortal database for CRC. The results shown in Figure 6C indicated a low to moderate correlation in the expression patterns among CXCL4/5/6/7/8/11/13/14/15/16/17; a moderate to high correlation in the expression of CXCL9 with CXCL11, and CXCL9 with CXCL12; and a high correlation in the expression of CXCL1 with CXCL2, CXCL1 with CXCL3, CXCL2 with CXCL3, CXCL9 with CXCL10, and CXCL10 with CXCL11.

Using GeneMANIA, we identified the relationships among CXCL1/2/3/4/5/6/7/8/9/10/11/12/13/14/16/17 at the gene expression level. As shown as in Figure 6D, several relationships were identified among CXCL1/2/3/4/5/6/7/8/9/10/11/12/13/14/16/17, including co-localization, co-expression, and genetic interactions. In addition, we analyzed the interactions among CXCL1/2/3/4/5/6/7/8/9/10/11/12/13/14/16/17 at the protein expression level using STRING. In total, 111 edges and 16 nodes were identified in resulting the network.

### Cellular functions and pathways of CXC chemokines in patients with CRC

As shown in Supplementary Figure 1, the functions of CXCL1/2/3/4/5/6/7/8/9/10/11/12/13/14/16/17 were predicted by analyzing the GO terms and KEGG pathways using DAVID. The GO enrichment terms were classified into three aspects of function: CC (3 items), MF (5 items), BP (10 items), and KEGG pathway (9 items). The CC analysis demonstrated a significant enrichment of genes related to the extracellular space, external side of plasma membrane, and extracellular region (Supplementary Figure 1A). The MF analysis revealed enrichment in chemokine activity, CXCR3 chemokine receptor binding, heparin binding, CXCR chemokine receptor binding, and receptor binding (Supplementary Figure 1B). In addition, BP was associated with cell-cell signaling, chemotaxis, regulation of cell proliferation, positive regulation of leukocyte chemotaxis, immune response, chemokine-mediated signaling pathway, inflammatory response, response to lipopolysaccharide, cell chemotaxis, and G-protein coupled receptor signaling pathway (Supplementary Figure 1C). As shown as Supplementary Figure 1D, we also found significant enrichment in KEGG pathways, including chemokine signaling pathway (Supplementary Figure 2A), cytokine-cytokine receptor interaction (Supplementary Figure 2B), legionellosis, Salmonella infection, toll-like receptor signaling pathway, NOD-like receptor signaling pathway, rheumatoid arthritis, pertussis, and TNF signaling pathway.

### Correlation analysis between CXC chemokine expression and immune infiltration levels in CRC

**Figure 7:**
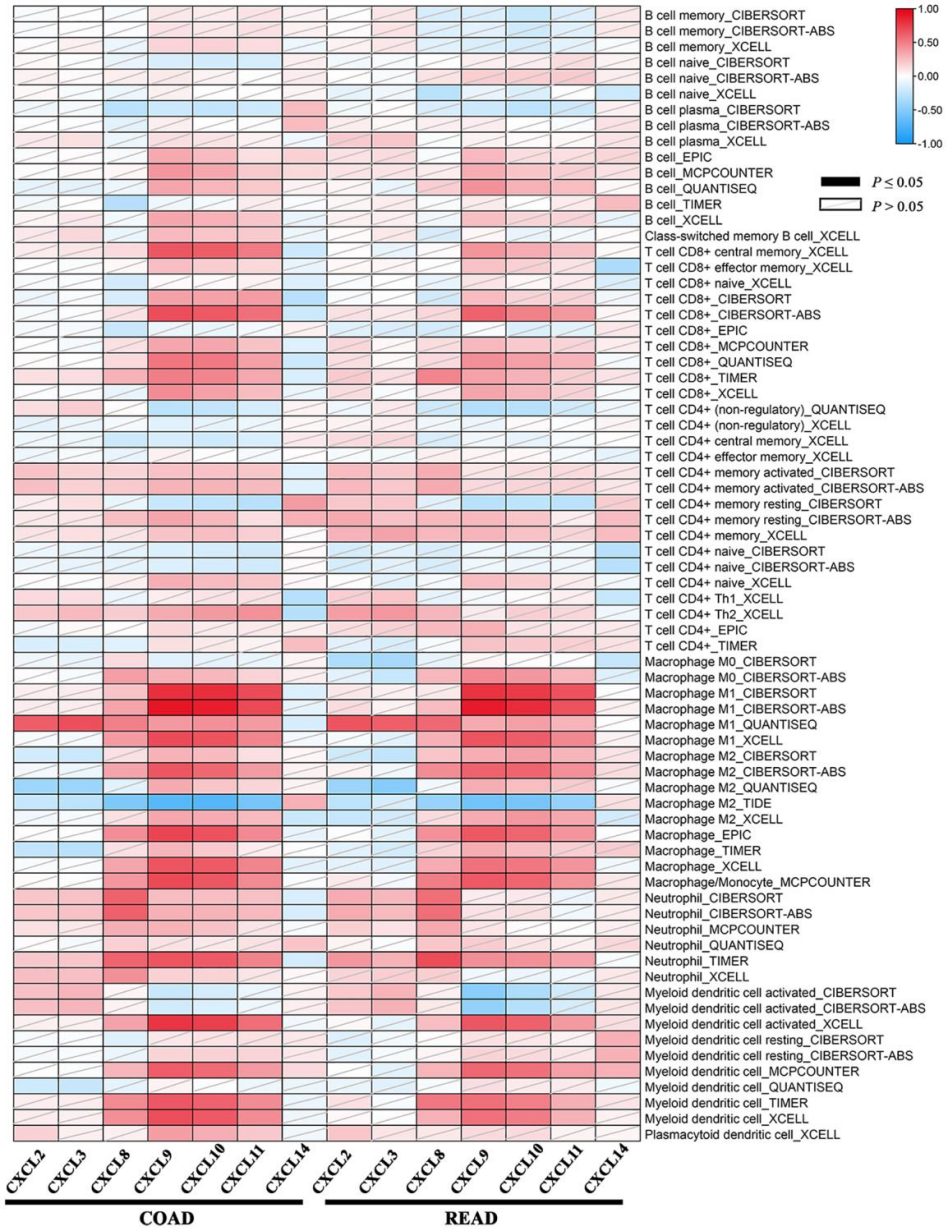
**The association between the CXC chemokines expression and immune infiltration level of multiple immune cells types estimated by all six algorithms in ad heatmap table across COAD and READ.** The red indicates a statistically significant positive association (*P* ≤ 0.05, rho > 0), and the blue indicates a statistically significant negative association (*P* ≤ 0.05, rho < 0). White denotes a non-significant result (*P* > 0.05).

Tumor-infiltrating lymphocytes are used to analyze the sentinel lymph node status and survival in cancers. According to TIMER2.0, we observed the correlation between the CXCL2/3/8/9/10/11/14 expression and the abundance of immune cell type as well as its subtypes estimated by all six algorithms across COAD and READ. As shown in Figure 7, the heatmap table represented the purity-adjusted partial spearman’s rho values as the degree of correlation. Moreover, a significant association was observed between the CXCL8/9/10/11 expression and immune infiltration in COAD and READ.

### Correlation analysis between CXC chemokine expression and immune markers

**Figure 8:**
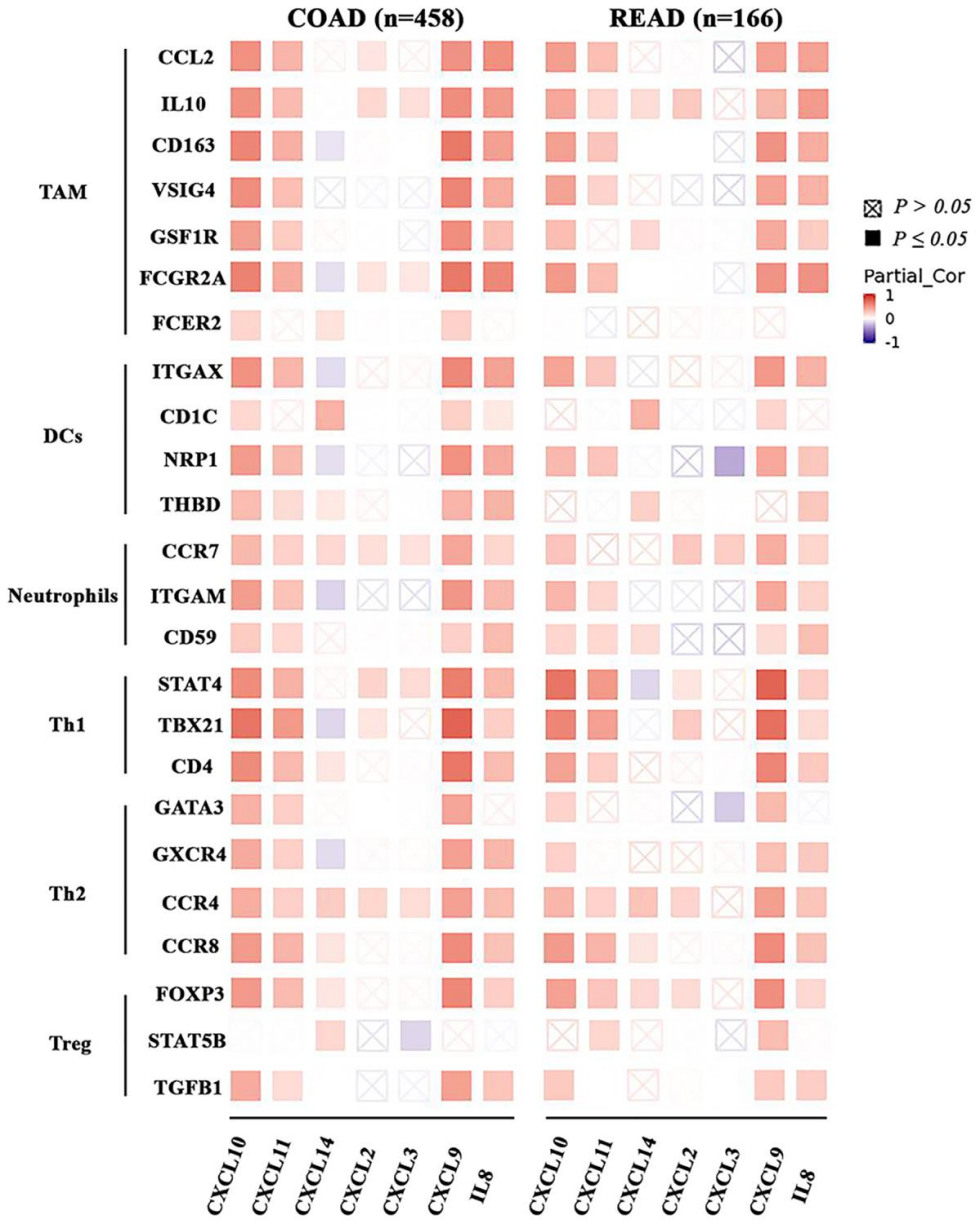
**The association between the CXC chemokines expression and immune markers in ad heatmap table across COAD and READ.** The red indicates a statistically significant positive association (*P* ≤ 0.05, rho > 0), and the blue indicates a statistically significant negative association (*P* ≤ 0.05, rho < 0). White denotes a non-significant result (*P* > 0.05).

To further investigate the association between the expression of CXCL2/3/8/9/10/11/14 and the diverse immune cell infiltration in CRC patients, we analyzed the relationship between CXC chemokines and sets of markers of various immune cells based on TIMER2.0, including tumor-associated macrophages (TAMs), neutrophils, DCs, and T helper 1 (Th1), Th2 and regulatory T (Treg) cells (Figure 8). Specifically, the CXCL 8/9/10/11 expression in COAD and READ was correlated with high levels of infiltration by TAMs, DCs, neutrophils, Th1, Th2 and Tregs. In addition, the results shown in Figure 8 confirmed the role of CXCL2/3/8/9/10/11/14 in the colorectal adenocarcinoma microenvironment.

## DISCUSSION

CXC chemokines have been reported to function as inflammatory mediators and drivers of leukocytes. Although the crucial role of CXC chemokines in tumor cell proliferation, tumorigenesis, tumor metastasis, and prognosis of several cancers has been partially elucidated, the biological functions, and prognostic value of these molecules in CRC remain to be fully characterized [3]. We used an integrative bioinformatics approach to investigate the mRNA expression, prognostic values (OS and DFS), biological functions, and correlations with immune cells of different CXC chemokines in CRC.

In the Oncomine database, we found that the expression levels of CXCL1/2/3/4/5/6/7/8/9/10/11/16/17 were significantly upregulated while that of CXCL12/13/14 were downregulated in CRC patients. These results were majorly consistent with our findings from the GEPIA database, which indicated that the CXCL1/2/3/4/5/8/9/10/11/13/14/16 expression was significantly upregulated in COAD and READ tissues relative to that in the normal tissue, while the CXCL12 expression was downregulated. Our results were also consistent with those reported previously. For example, Zhuo et al. reported that the higher CXCL1 expression correlated with cancer progression and metastasis in CRC patients [15]. Mechanistically, the knockdown of CXCL1 could reduce the levels of some glycolytic enzymes (HK2, GLUT1, and LDHA). In addition, Doll et al. reported that the expression of CXCL2, CXCL3, and CXCL8 in the colon cancer tissues was significantly higher than that in the normal tissues [16]. Zhao et al. also demonstrated that CXCL5 was upregulated in the tumor tissues, which was associated with poor prognosis and advanced tumor stage in CRC patients [17]. Moreover, Zhang et al. discovered that CXCL5 was highly expressed in CRC, and that its overexpression could reverse the suppressive effect of miR-363-3p on CRC progression [18]. Overall, CXCL1/2/3/4/5/8/9/10/11/13/14/16 are highly expressed in CRC patients, thereby serving as an oncogene in CRC in addition to their association with prognosis.

Several past studies have reported a correlation between CXC chemokines expression and the prognosis of CXC patients. For example, Xiong et al. noted that the high expression of CXCL3 was closely associated with poor OS in CRC patients from the TCGA dataset [19]. Survival analysis conducted on GEO cohorts and Guangxi Medical University revealed that the high CXCL3 expression levels were associated with a shorter OS in colon cancer patients [20]. However, Doll et al. recorded no significant correlation between the CXCL3 expression levels and CRC survival [16]. Wu et al. also reported that the higher of CXCL9 mRNA and protein expression was significantly associated with OS of CRC patients [21]. Zeng et al. reported that the elevated CXCL14 expression in the CRC tissues from stage III/IV patients was associated with worse OS, thus representing a potential therapeutic target in CRC patients after curative resection [22]. However, other research groups have indicated that high levels of CXCL14 are correlated with a good prognosis [12]. Matsushita et al. reported that the high levels of preoperative serum levels of soluble CXCL16 in CRC patients were associated with a poor prognosis [23]. Due to the different sources of CRC patients, different studies recorded varied expression levels of CXC chemokines within the CRC tissues. In the present study, we analyzed the correlation between the CXC chemokine expression and the OS and DFS of patients with CRC using GEPIA. The results demonstrated that the high mRNA expression levels of CXCL2/3/8/14 were correlated with longer OS in CRC patients. In addition, the increased mRNA expression levels of CXCL9/10/11 were associated with longer DFS in CRC patients. Moreover, the expression of CXCL1/2/3/9/10/11 was associated with the tumor stage in CRC. A significant association was also identified for the co-expression of CXCL16 with EGFR, KRAS and NRAS. These results together suggest that CXC chemokines act as potential biomarkers for predicting CRC prognosis. Nevertheless, these results should be investigated in future studies using *in vitro* and *in vivo* experimental setting for validation.

The mechanisms underlying the role of CXC chemokines on CRC remain complex. For instance, Chen et al. found that metastasis suppressor 1 (MTSS1) may play an important suppressive role in CRC metastasis and that the underlying mechanisms may involve the downregulation of the CXCR4/CXCL12 signaling axis [24]. Zhang et al. discovered that CXCL5 was highly expressed in CRC, and that circCTNNA1 could act as a ceRNA for miR-363-3p to facilitate the progression of CRC through the promotion of the CXCL5 expression [18]. Zhao et al. found that the overexpression of CXCL5 could induce the epithelial-mesenchymal transition (EMT) to enhance the invasion and migration of CRC cells by the activation of the AKT/GSK3β/β-catenin and the ERK/Elk-1/Snail pathways in a CXCR2-dependent manner [17]. Xia et al. indicated that cisatracurium could suppress the viability, metastasis, and tumor growth of CRC by regulating the CXCR4/let-7a-5p axis through the inhibition of the TGF-β/SMAD2/3 signaling pathway [25]. GO and KEGG enrichment analyses were conducted for the CXC chemokines to evaluate their biological functions of them. In line with previous results, we found that the most-enriched GO terms were the chemokine activity, CXCR3 chemokine receptor binding, CXCR chemokine receptor binding, chemotaxis, regulation of cell proliferation, positive regulation of leukocyte chemotaxis, immune response, chemokine-mediated signaling pathway, inflammatory response, cell chemotaxis, and G-protein coupled receptor signaling pathway. The most commonly enriched KEGG pathways were the chemokine signaling pathway, cytokine-cytokine receptor interaction, toll-like receptor signaling pathway, NOD-like receptor signaling pathway, rheumatoid arthritis, pertussis, and the TNF signaling pathway. Therefore, further investigations are warranted to comprehend the biological functions of CXC chemokine.

Moreover, we identified a significant association between the CXCL2/3/8/9/10/11/14 expression and immune infiltration in CRC using TIMER2.0. CXCL8 plays an important role in inflammatory responses, leukocyte chemotaxis, and infectious diseases [11, 26, 27]. Li et al. reported that targeting the CXCL8-CXCR1/CXCR2 axis can act as a key factor in mediating the antitumor effects on CRC by impeding DC activation or recruitment [28]. In line with the results of some previous studies, we demonstrated that the CXCL8 expression was upregulated. Moreover, the CXCL8 expression level in COAD and READ was significantly and positively associated with infiltration by neutrophils, macrophage, and DCs. CXCL9/10/11 exerted their biological effects related to EMT via CXCR3, which was upregulated in the invasive front of the CRC tumor tissues [29]. CXCL9/10/11 and CXCR3 play pivotal roles in the activation, differentiation, and effector T cell function. CXCR3 is important for CD8 effectors, CD4 Th1 cells, memory cells, and for the functioning of natural killer T cells and natural killer in the CRC tissues. Our study also revealed that CXCL9/10/11 was overexpressed, and positively associated with PFS of CRC patients. More importantly, infiltration by immune cells, including CD8+ T cells, neutrophils, macrophages, and DCs, was found to be associated with the CXCL9/10/11 expression. Thus, these results together indicate that CXCLs are closely related to the immune infiltration of CRC, thereby providing new concepts for immunotherapy.

CXC chemokines not only serve as prognostic biomarkers and participate in the regulation of the tumor microenvironment, but also represent the potential therapeutic targets for CRC patients. It has been reported that the activation of the CXCR1/CXCR2 or CXCR4/CXCR7 pathways is associated with tumor aggressiveness and poor prognosis. Therefore, specific inhibition of these receptors can be considered as a possible therapeutic strategy [30]. For example, Wang et al. found that prostaglandin E2 (PGE2) induced of CXCL1 expression, thus implicating CXCL1 inhibitors as potential anti-angiogenic agents in the treatment of CRC [31]. Liu et al. discovered that the LPS-induced CXCR4 expression and EMT activation via the NF-κB signaling pathway promoted CRC progression [32]. Liao et al. indicated that the anti-PD-1 resistance of KRAS-expressing tumors could be overcome to some extent by inhibiting the CXCR2 expression [33]. Ning et al. revealed that CXCL8 regulated OXA-resistance of CRC cell lines by binding to CXCR2 and activating the AKT/MAPK/NF-κB signaling pathway [11]. Jamieson et al. reported that CXCR2 antagonists exert therapeutic effects in a mouse model of colon cancer [34]. Murakami et al. found that targeting CXCR3 and CXCR4 inhibited CRC metastasis to the liver and lung [35]. Moreover, Rupertus et al. indicated that CXCL11 and CXCL12 may play important roles in tumor angiogenesis, and blocking their expression inhibited tumor angiogenesis and growth [36]. In addition, the CXCL4/9/10/11 expression levels were closely associated with chemotherapy and radiation therapy in CRC patients [37–39]. However, the role of other CXC chemokines, such as CXCL16/17, remains unclear. Overall, although the studies on CXC chemokines as therapeutic targets in CRC are presently preliminary, with ensuing researches, their roles in the treatment of CRC are expected to become increasingly clear, thereby improving CRC patient survival by targeting CXC chemokines.

## CONCLUSION

In this study, we conducted a systematic analysis of the expression, prognostic value, and signaling pathways of CXC chemokines, to provide a comprehensive understanding of the molecular biological properties of CRC. The mRNA expression levels of CXCL1/2/3/4/5/8/9/10/11/13/14/16 were significantly elevated in CRC tissues and we observed a significant correlation between the expression of CXCL1/2/3/9/10/11 and the tumor stage in CRC. Moreover, we also identified a significant association between the co-expression of CXCL16 with EGFR, KRAS and NRAS. Our findings suggest that the high CXCL2/3/8/9/10/11/14 expression is correlated with the clinical outcomes CRC patients. Interestingly, a significant correlation was identified between CXCL8/9/10/11 expression and tumor infiltration by immune cell types. Overall, CXC chemokines are not only valuable prognostic markers for CRC patients, but may also influence immune status of CRC tissues.

## Supplementary Materials

Supplementary Figures

Supplementary Tables
