# Supplementary material for: Comprehensive analysis of the prognosis and immune infiltration for CXC chemokines in colorectal cancer
Source: Aging (Albany NY). 2021 Jul 7;13(13):17548–67. doi: 10.18632/aging.203245 (PMC8312455; doi:10.18632/aging.203245)
Supplement: Supplementary Figures [file aging-13-203245-s001.pdf]

SUPPLEMENTARY FIGURES

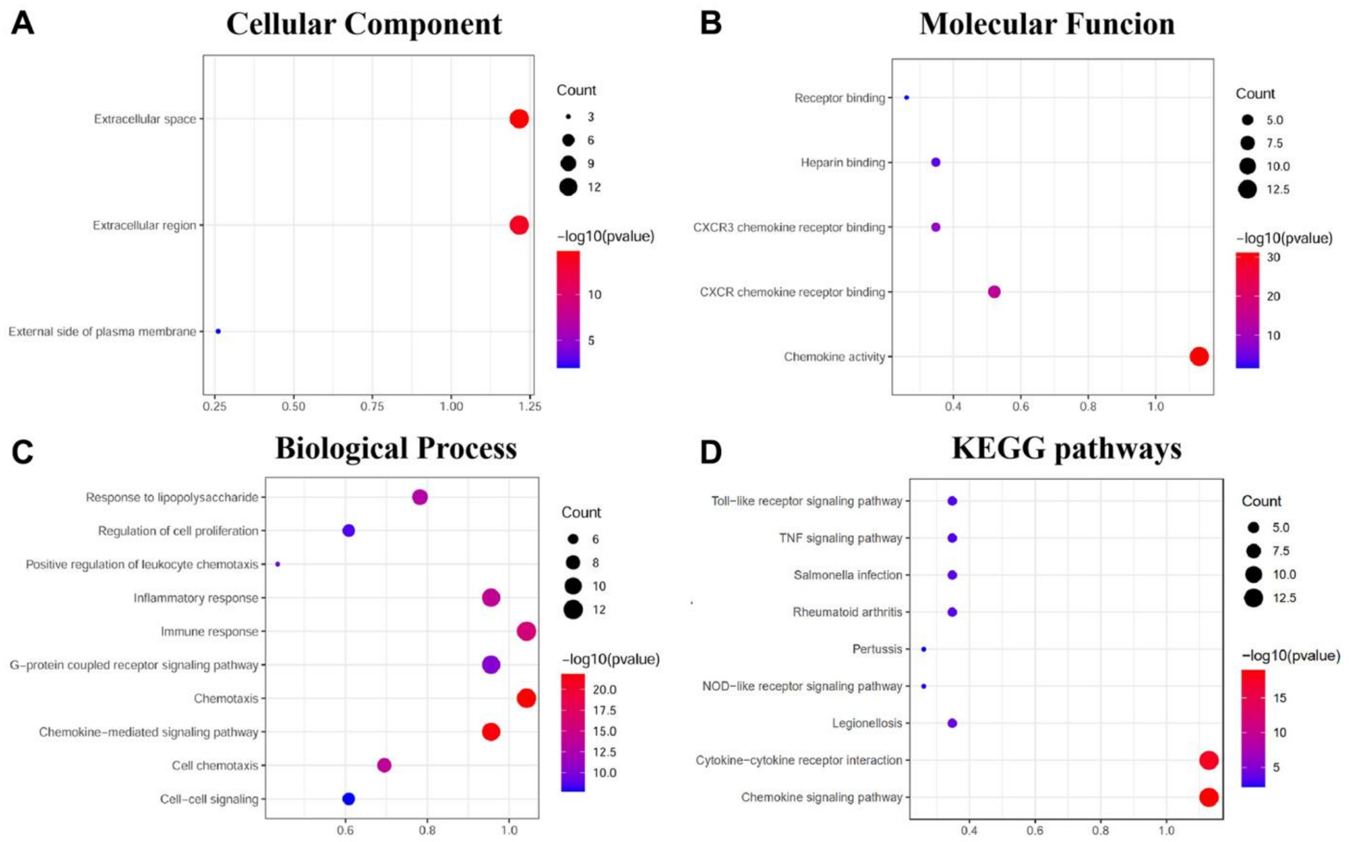

Supplementary Figure 1. The enrichment analysis of different expressed CXC chemokines in CRC (David 6.8), including (**A**) cellular components (CC), (**B**) molecular functions (MF), (**C**) biological processes (BP), and (**D**) KEGG enriched terms.

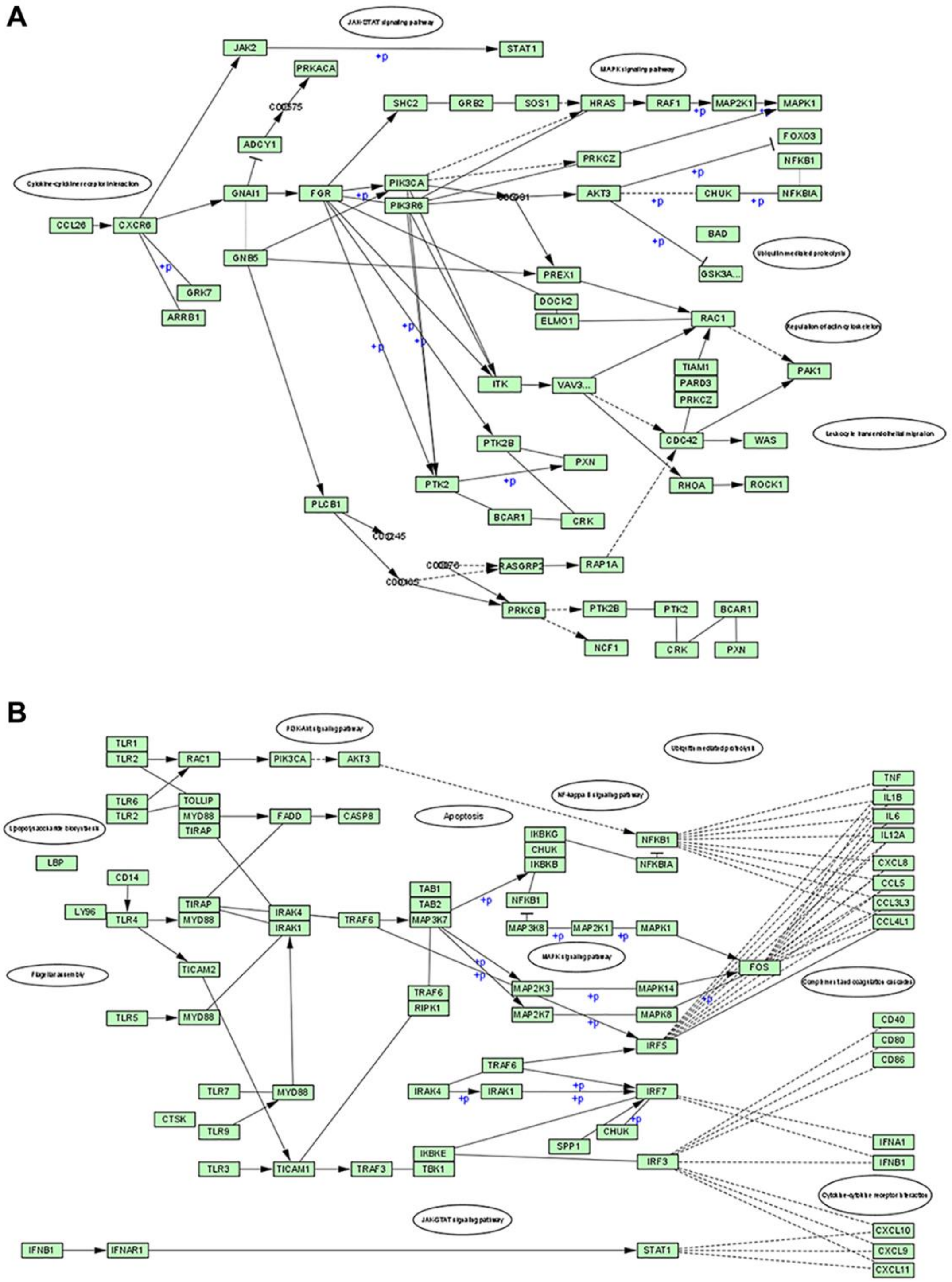

Supplementary Figure 2. (**A**) Chemokine signaling pathway and (**B**) toll-like receptor signaling regulated by the CXC chemokines in CRC.
