# Supplementary material for: Comprehensive analysis of the prognosis and immune infiltration for CXC chemokines in colorectal cancer
Source: Aging (Albany NY). 2021 Jul 7;13(13):17548–67. doi: 10.18632/aging.203245 (PMC8312455; doi:10.18632/aging.203245)
Supplement: Supplementary Tables [file aging-13-203245-s002.pdf]

SUPPLEMENTARY TABLES

Supplementary Table 1. Correlation between different expressed CXC chemokines and the tumor stage of CRC patients (GEPIA).

	P (>F)		
	COAD + READ	COAD	READ
**CXCL1**	**0.0345**	0.287	0.0523
**CXCL2**	**0.0156**	0.131	**0.0371**
**CXCL3**	**0.0344**	0.316	**0.00868**
**CXCL4**	0.645	0.945	0.422
**CXCL5**	0.768	0.783	0.615
**CXCL6**	0.287	0.545	0.299
**CXCL7**	0.519	0.377	0.902
**CXCL8**	0.417	0.665	0.598
**CXCL9**	**0.0122**	**0.00267**	0.971
**CXCL10**	**0.0108**	**0.00584**	0.895
**CXCL11**	**0.0227**	**0.0384**	0.888
**CXCL12**	0.881	0.738	0.361
**CXCL13**	0.217	0.169	0.918
**CXCL14**	0.28	0.239	0.108
**CXCL16**	0.274	0.165	0.96
**CXCL17**	0.59	0.68	0.621

Abbreviations: COAD: colon adenocarcinoma; READ: rectum adenocarcinoma.

Supplementary Table 2. The prognostic value of different expressed CXC chemokines in CRC patients in OS and DFS (GEPIA).

	Logrank *P* (OS)			Logrank *P* (DFS)		
	COAD + READ	COAD	READ	COAD + READ	COAD	READ
**CXCL1**	0.06	0.13	0.41	0.49	0.34	0.73
**CXCL2**	**0.052**	**0.042**	0.49	0.35	0.49	0.2
**CXCL3**	**0.015**	**0.047**	0.17	0.51	0.46	0.83
**CXCL4**	0.34	0.52	0.018	0.43	0.42	0.15
**CXCL5**	0.62	0.68	0.9	0.079	0.21	0.27
**CXCL6**	0.11	0.44	0.068	0.12	0.35	0.15
**CXCL7**	0.66	0.22	0.13	0.14	0.084	0.78
**CXCL8**	**0.032**	**0.05**	0.96	0.28	0.41	0.67
**CXCL9**	0.45	0.55	0.38	**0.059**	**0.026**	0.66
**CXCL10**	0.47	0.56	0.29	**0.0019**	**0.0011**	0.64
**CXCL11**	0.61	0.54	0.32	**0.0096**	**0.0045**	0.57
**CXCL12**	0.4	0.68	0.91	0.086	0.46	0.099
**CXCL13**	0.24	0.61	0.13	0.52	0.27	0.97
**CXCL14**	**0.039**	**0.045**	0.18	0.14	0.16	0.69
**CXCL16**	0.15	0.11	0.52	0.37	0.18	0.54
**CXCL17**	0.95	0.94	0.11	0.78	0.78	0.99

Abbreviations: OS: overall survival; DFS: disease free survival; COAD: colon adenocarcinoma; READ: rectum adenocarcinoma.
